# Synthesis and Antimycobacterial Activity of some Triazole Derivatives–New Route to Functionalized Triazolopyridazines

**Published:** 2015

**Authors:** Kamaleddin Haj Mohammad Ebrahim Tehrani, Vida Mashayekhi, Parisa Azerang, Somayeh Minaei, Soroush Sardari, Farzad Kobarfard

**Affiliations:** a *Department of Medicinal Chemistry, School of Pharmacy, Shahid Beheshti University of Medical Sciences, Tehran, Iran. *; b *Drug Design and Bioinformatics Unit, Department of Medical Biotechnology, Biotechnology Research Center, Pasteur Institute; Tehran, Iran. *; c *Phytochemistry Research Center, Shahid Beheshti University of Medical Sciences, Tehran, Iran.*

**Keywords:** Triazole, Triazolopyridazine, Thiocarbohydrazide, Antimycobacterial

## Abstract

A series of cyclic analogues of bioactive thiosemicarbazide derivatives have been synthesized as potential antimycobacterial agents. The 4-amino-1,2,4-triazole-5-thione analogues (Ia-f) were prepared by heating a mixture of thiocarbohydrzide and appropriate carboxylic acids. Reaction of thiocarbohydrazide with γ-ketoesters in the presence of sodium methoxide furnished triazolopyridazine derivatives IIa-b. Finally, condensation of 4-amino-1,2,4-triazole-5-thione with some aldehydes gave Schiff bases IIIa-e. After characterization by different spectroscopic and analytical methods, the derivatives were tested for their inhibitory activity against *Mycobacterium bovis BCG*. Among the derivatives, compound Ib proved to be the most potent derivatives with MIC value of 31.25 µg/mL. Given the fact that 4-amino-1,2,4-triazole-5-thiones Ia-f were the most active derivatives, it could be suggested that this group of derivatives have the potential to be considered as lead compounds for future optimization efforts.

## Introduction

From the standpoint of synthetic organic chemistry, thiosemicarbazide (TSC) and thiocarbohydrazide (TCH) are interesting molecules due to various organic transformations in which they can take part. Their sulfur atom as an analogue of thiourea is highly nucleophilic toward C-electrophiles and the terminal amino group due to the activating effect of its adjacent nitrogen is prone to participate in diverse reactions including nucleophilic substitution, amide formation and Schiff base formation (1). Owing to these two reactive sites, TSC and TCH have been reported to form diverse five, six and seven membered heterocycles upon treatment with different substrates such as carboxylic acids or their synthetic equivalents (esters, orthoesters or acid chlorides), *α*,*β*-unsaturated carbonyl compounds, *β*-dicarbonyls and halomethylketones (2-6). On the other hand, when incorporated in the organic molecules of pharmacological interest, they provide a “synthetic handle” in the molecule which enables the medicinal chemist to directly manipulate these bioactive molecules during another organic transformation. The latter is a favorite strategy in drug discovery efforts and is known as lead expansion (7). 

TSC and TCH derivatives have been reported to possess various biological properties including antimicrobial (8-10), antimycobacterial (11-13), antiprotozoal (14) and anticancer (15,16) activities. Recently, as a part our focused studies on the antimycobacterial activities of Schiff bases of TSC and TCH, we found some novel cyclic analogues of antimycobacterial thiosemicarbazones with satisfactory activity against *Mycobacterium bovis BCG*. In addition we found new synthetic application of TCH which was formation of Schiff base and ring closure to thiadiazole in a one-pot methodology (17). In continuation of our interest to discover cyclic analogues of TCH with acceptable antimycobacterial activity, in the present study we have described the synthesis and bioactivity of some triazole derivatives and also first synthesis of novel fused 6-aryl-7,8-dihydro-[1,2,4]triazolo[4,3-*b*]pyridazines.

**Figure 1:**
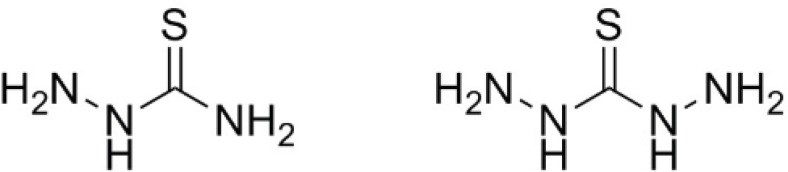
Structures of thiosemicarbazide (left) and thiocarbohydrazide (right).

## Experimental

*General*

 The reactions progress were monitored using TLC silica gel 60 F254 plates (Merck) with chloroform/methanol as mobile phase. Melting points were measured by an Electrothermal 9100 apparatus and are uncorrected. Infrared spectra were obtained by a Perkin-Elmer 843 spectrometer. Proton nuclear magnetic resonance (^1^H NMR) spectra and carbon nuclear magnetic resonance (^13^C NMR) spectra were determined on a Bruker Avance DRX 400 MHz spectrometer and chemical shift values have been reported as *δ* (ppm) in DMSO-*d*_6_ solution (0.05% v/v TMS). ESI-MS spectra were obtained using Agilent 6410 Triple Quad. LC/MS. All the compounds were analyzed for C, H, N and S on a Costech model 4010 and agreed with the proposed structures within ±0.4% of the theoretical values.

*Synthesis of 3-substituted 4-amino-1,2,4-triazole-5-thione derivatives (Id-f)*

Equimolar amounts of thiocarbohydrazide and appropriate carboxylic acids (10 mmol of each) were mixed and heated at 165-170 °C for 30 min. Boiling water (20 mL) was added to the solid and the mixture was kept at room temperature for 24 h. The precipitate was filtered and recrystallized from ethanol to afford the title compounds.

*4-amino-3-(indol-3-ylmethyl)-1,2,4-triazole-5-thione (Id)*

 Yellow solid, yield 57%, mp 172-175 °C; IR ν_max_(KBr)/cm^-1^ 3304, 3150, 1615, 1567, 1494, 1336, 1098, 944, 753; ^1^H NMR (400 MHz, DMSO-*d*_6_) *δ* 4.12 (s, 2H, CH_2_), 5.59 (br s, 2H, NH_2_), 6.99 (t, 1H, *J* = 7.2 Hz, indole H-5), 7.09 (t, 1H, *J* = 7.4 Hz, indole H-6), 7.26 (s, 1H, indole H-2), 7.36 (d, 1H, *J* = 8.0 Hz, indole H-7), 7.54 (d, 1H, *J* = 8.0 Hz, indole H-4), 10.98 (s, 1H, NH), 13.48 (s, 1H, NH); ESI-MS *m/z*: 246 (M + H^+^). Anal. Calcd for C_11_H_11_N_5_S: C, 53.86; H, 4.52; N, 28.55; S, 13.07. Found: C, 53.95; H, 4.53; N, 28.61; S, 13.03.

*4-amino-3-(hydroxy(phenyl)methyl)-1,2,4-triazole-5-thione (Ie)*

 white solid, yield 43%, mp 167-170 °C; IR ν_max_(KBr)/cm^-1^ 3293, 3178, 1617, 1553, 1479, 1189, 759, 694; ^1^H NMR (400 MHz, DMSO-*d*_6_) *δ* 5.59 (2 close singlets, 2H, NH_2_), 5.89 (d, 1H, *J* = 5.4 Hz, -C*H*-OH), 6.33 (d, 1H, *J* = 5.4 Hz, D_2_O exchangeable, OH), 7.31-7.39 (m, 3H, Ar H), 7.44 (d, 2H, *J* = 8.0 Hz, Ar H), 13.60 (s, 1H, NH); ESI-MS *m/z*: 223 (M + H^+^). Anal. Calcd for C_9_H_10_N_4_OS: C, 48.63; H, 4.53; N, 25.21; S, 14.43. Found: C, 48.49; H, 4.54; N, 25.13; S, 14.48.

*4-amino-3-(thiophen-2-ylmethyl)-1,2,4-triazole-5-thione (If)*

Yellow solid, yield 51%, mp 154-157 °C; IR ν_max_(KBr)/cm^-1^ 3295, 3166, 1631, 1491, 1335, 940, 695; ^1^H NMR (400 MHz, DMSO-*d*_6_) *δ* 4.26 (s, 2H, CH_2_), 5.58 (2 close singlets, 2H, NH_2_), 6.99 (m, 2H, thiophene H-3,4), 7.42 (dd, 1H, *J* = 5.2 Hz, *J* = 1.2 Hz, thiophene H-5), 13.60 (s, 1H, NH); ESI-MS *m/z*: 213 (M + H^+^). Anal. Calcd for C_7_H_8_N_4_S_2_: C, 39.60; H, 3.80; N, 26.39; S, 30.21. Found: C, 39.48; H, 3.79; N, 26.45; S, 30.12.

*Synthesis of 6-Aryl-7,8-dihydro-[1,2,4]triazolo[4,3-b]pyridazine-3-thione derivatives (IIa-b)*

In a 50 mL flask connected to a calcium chloride guard tube, sodium (104 mg, 4.5 mmol) was dissolved in anhydrous methanol (15 mL). Thiocarbohydrazide (480 mg, 4.5 mmol) was added and the mixture was heated under reflux for 5 min. Appropriate γ-ketoesters (each 4.5 mmol) were then added and the mixture was further heated under reflux for 24 h. The flask was then surrounded by ice-water and the mixture was neutralized by dilute hydrochloric acid which led to precipitation of the products. The solid was filtered off, washed with n-hexane and diethyl ether and dried to afford the triazolopyridazines IIa and IIb.

*6-phenyl-7,8-dihydro-[1,2,4]triazolo[4,3-b]pyridazine-3-thione (IIa)*

White solid, yield 39%, mp 253 °C (dec.); IR ν_max_(KBr)/cm^-1^ 3309, 1604, 1588, 1366, 1334, 1014, 759, 685; ^1^H NMR (400 MHz, DMSO-*d*_6_) *δ* 3.10 (m, 4H, methylene H), 7.54 (m, 3H, Ar H-3,4,5), 7.97 (d, 2H, *J* = 7.0 Hz, Ar H-2,6), 13.67 (s, 1H, NH); ESI-MS *m/z*: 231 (M + H^+^), 253 (M + Na^+^). Anal. Calcd for C_11_H_10_N_4_S: C, 57.37; H, 4.38; N, 24.33; S, 13.92. Found: C, 57.28; H, 4.39; N, 24.40; S, 13.91.

*6-(4-chlorophenyl)-7,8-dihydro-[1,2,4]triazolo[4,3-b]pyridazine-3-thione (IIb)*

Yellow solid, yield 35%, mp 256 °C (dec.); IR ν_max_(KBr)/cm^-1^ 3115, 1605, 1585, 1483, 1370, 1091, 837, 783; ^1^H NMR (400 MHz, DMSO-*d*_6_) *δ* 3.09-3.16 (m, 4H, methylene H), 7.63 (d, 2H, *J* = 6.8 Hz, Ar H), 8.00 (d, 2H, *J* = 6.8 Hz, Ar H), 13.67 (s, 1H, NH); ESI-MS *m/z*: 265, 267 (M + H^+^). Anal. Calcd for C_11_H_9_ClN_4_S: C, 49.91; H, 3.43; N, 21.16; S, 12.11. Found: C, 50.05; H, 3.42; N, 21.09; S, 12.13.

*Synthesis of Schiff bases of 4-amino-1,2,4-triazole-5-thione (IIIa-e)*

Equimolar amounts of 4-amino-1,2,4-triazole-5-thione (Ia) and appropriate aldehydes (3 mmol of each) in glacial acetic acid (5 mL) were heated at 90 °C for 20 min. The mixture was then cooled to room temperature, filtered and washed with water (3 × 20 mL). The obtained crudes were recrystallized from ethanol to afford the Schiff bases IIIa-e.

*4-(pyridin-2-ylmethyleneamino)-1,2,4-triazole-5-thione (IIIa)*

Yellow solid, yield 67%, mp 213-216 °C; IR ν_max_(KBr)/cm^-1^ 3108, 1617, 1585, 1574, 1492, 1333, 1208, 938, 784; ^1^H NMR (400 MHz, DMSO-*d*_6_) *δ* 7.58 (dd, 1H, *J* = 7.6 Hz, *J* = 4.7 Hz, pyridine H-5), 8.00 (t, 1H, *J* = 7.6 Hz, pyridine H-4), 8.11 (d, 1H, *J* = 7.9 Hz, pyridine H-3), 8.7 (d, 1H, *J* = 4.7 Hz, pyridine H-6), 9.03 (s, 1H, triazole H), 9.64 (s, 1H, imine H), 13.99 (s, 1H, NH); ^13^C NMR (100 MHz, DMSO-*d*_6_) *δ* 121.5, 126.2, 137.3, 138.2, 150.0, 151.2, 158.8, 163.1; ESI-MS *m/z*: 206 (M + H^+^). Anal. Calcd for C_8_H_7_N_5_S: C, 46.82; H, 3.44; N, 34.12; S, 15.62. Found: C, 46.95; H, 3.43; N, 34.22; S, 15.58.

*4-(pyridin-3-ylmethyleneamino)-1,2,4-triazole-5-thione (IIIb)*

Yellow solid, yield 59%, mp 157 °C (dec.); IR ν_max_(KBr)/cm^-1^ 3123, 1600, 1505, 1428, 1307, 1203, 1186, 924, 881, 706; ^1^H NMR (400 MHz, DMSO-*d*_6_) *δ* 7.60 (dd, 1H, *J* = 7.9 Hz, *J* = 4.8 Hz, pyridine H-5), 8.28 (td, 1H, *J* = 7.9 Hz, *J* = 1.9 Hz, pyridine H-6), 8.77 (dd, 1H, *J* = 4.8 Hz, *J* = 1.7 Hz, pyridine H-4), 8.95 (s, 1H, triazole H), 8.98 (d, 1H, *J* = 1.9 Hz, pyridine H-2), 9.59 (s, 1H, imine H), 13.98 (s, 1H, NH); ^13^C NMR (100 MHz, DMSO-*d*_6_) *δ* 124.39, 128.29, 134.87, 138.07, 150.00, 152.92, 158.28, 163.07; ESI-MS *m/z*: 206 (M + H^+^). Anal. Calcd for C_8_H_7_N_5_S: C, 46.82; H, 3.44; N, 34.12; S, 15.62. Found: C, 46.91; H, 3.45; N, 34.01; S, 15.60.

*4-(pyridin-4-ylmethyleneamino)-1,2,4-triazole-5-thione (IIIc)*

Yellow solid, yield 71%, mp 255-256 °C; IR ν_max_(KBr)/cm^-1^ 3151, 1597, 1577, 1491, 1314, 1203, 1115, 931, 819; ^1^H NMR (400 MHz, DMSO-*d*_6_) *δ* 7.79 (d, 2H, *J* = 5.8 Hz, pyridine H-3,5), 8.78 (d, 2H, *J* = 5.8 Hz, pyridine H-2,6), 8.98 (s, 1H, traizole H), 9.59 (s, 1H, imine H), 14.01 (s, 1H, NH); ^13^C NMR (100 MHz, DMSO-*d*_6_) *δ* 121.75, 137.95, 139.29, 150.64, 157.65, 163.29; ESI-MS *m/z*: 206 (M + H^+^). Anal. Calcd for C_8_H_7_N_5_S: C, 46.82; H, 3.44; N, 34.12; S, 15.62. Found: C, 46.79; H, 3.45; N, 34.19; S, 15.67.

*4-(4-acetamidobenzyldeneamino)-1,2,4-triazole-5-thione (IIId)*

White solid, yield 75%, mp 271 °C (dec.); IR ν_max_(KBr)/cm^-1^ 3281, 3239, 3168, 1674, 1604, 1517, 1279, 956, 832; ^1^H NMR (400 MHz, DMSO-*d*_6_) *δ* 2.09 (s, 3H, CH_3_), 7.76 (d, 2H, *J* = 8.8 Hz, aromatic H-3,5), 7.81 (d, 2H, *J* = 8.8 Hz, aromatic H-2,6), 8.87 (s, 1H, triazole H), 9.32 (s, 1H, imine H), 10.29 (s, 1H, NH), 13.89 (s, 1H, NH); ^13^C NMR (100 MHz, DMSO-*d*_6_) *δ* 24.06, 118.83, 126.40, 129.48, 138.27, 143.02, 160.96, 162.63, 168.77; ESI-MS *m/z*: 262 (M + H^+^). Anal. Calcd for C_11_H_11_N_5_OS: C, 50.56; H, 4.24; N, 26.80; S, 12.27. Found: C, 50.71; H, 4.23; N, 26.72; S, 12.26.

*4-((E)-3-phenylallylideneamino)-1,2,4-triazole-5-thione (IIIe)*

Yellow solid, yield 95%, mp 185 °C (dec.); IR ν_max_(KBr)/cm^-1^ 3150, 1660, 1539, 1201, 1125, 1007, 987, 771, 693; ^1^H NMR (400 MHz, DMSO-*d*_6_) *δ* 7.20 (dd, 1H, *J* = 16.0 Hz, *J* = 9.5 Hz, Ph-CH=C*H*-CH=N-), 7.38 (d, 1H, *J* = 16.0 Hz, Ph-C*H*=CH-CH=N-), 7.43 (m, 3H, phenyl H-3,4,5), 7.74 (dd, 2H, *J* = 6.5 Hz, *J* = 1.5 Hz, phenyl H-2,6), 8.85 (s, 1H, triazole H), 9.23 (d, 1H, *J* = 9.5 Hz, Ph-CH=CH-C*H*=N-), 13.89 (s, 1H, NH); ^13^C NMR (100 MHz, DMSO-*d*_6_) *δ* 123.59, 127.98, 129.01, 130.17, 134.98, 138.05, 145.89, 162.45, 162.78; ESI-MS *m/z*: 231 (M + H^+^). Anal. Calcd for C_11_H_10_N_4_S: C, 57.37; H, 4.38; N, 24.33; S, 13.92. Found: C, 57.48; H, 4.39; N, 24.42; S, 13.88.

*In-vitro evaluation of antimycobacterial activity*

The synthesized derivatives were assayed for their antimycobacterial activity against *Mycobacterium bovis BCG* (1173P2) by microtiter broth dilution method according to our previous works (13,17,18). Briefly, 100 µL of freshly prepared Middle broke 7H9 medium was added to all the wells of microplates, except the first column which received 200 µL of distilled water. Then 100 µL of test compounds with desired concentrations were added to the wells of the first row (each concentration was assayed in duplicate) and serial dilution was made from the first row to the last. Microbial suspension of BCG (1173P2, 100 µL) with standard concentration of 0.5 Mcfarland was diluted with 1:10 proportion by the distilled water and added to all test wells. Plates were then sealed and incubated for 4 days at 37 °C. Then, 12 µL of 10% Tween 80 and 20 µL of 0.01% Alamar blue (Himedia, India) were added to each test well. The results were assessed after 24 and 48 hours. A blue color was interpreted as no bacterial growth, and color change to pink was scored as bacterial growth. Wells with a well-defined pink color were scored as positive for growth. The MIC (minimal inhibition concentration) was defined as the lowest drug concentration, which prevented a color change from blue to pink. Ethambutol and DMSO were used as positive and negative control respectively.

*Cytotoxicity assay*

The synthesized derivatives were assayed for their toxicity on Fibroblast L929 cell line by MTT [3-(4,5-dimethylthiazol-2-yl-2,5-tetrazolium bromide)] method. The cells were grown in RPMI1640 medium at 37 °C under 5% CO_2_ supplemented with 10% heat inactivated fetal bovine serum (FBS), 100 U/mL penicillin and 100 µg/mL streptomycin. Then, the cells were seeded into 96-well plates at a concentration of 6000 cells/well and allowed to incubate for 24 h. The medium was then discarded and different concentrations of test compounds in complete medium were added to each well. After further incubation for 24 h at 37 °C, the medium was discarded and 100 µL MTT (2 mg/mL) was added to the wells and incubated for 3 h at 37 °C. The produced formazan crystals were dissolved in 100 µL of DMSO. Plates were incubated for 20 min at 37 °C and the optical densities were read at 570 nm with a reference wavelength of 630 nm as background using a spectrophotometer plate reader (Infinite® M200, TECAN). Doxorubicin and cisplatin were used as positive controls and DMSO as the solvent of the test compounds and its final concentration was less than 0.2%. IC_50_ was calculated by GraphPad Prism 5.04 software. All the tests were performed in triplicates.

## Results and Discussion

*Chemistry*

**Figure 2:**
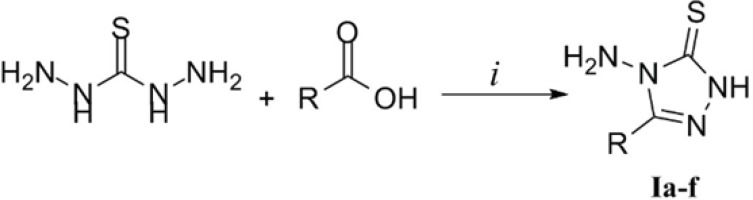
Synthesis of compounds Ia-f. Reaction conditions: *i*) heat to reflux (for Ia-c) or heat at 164-170 °C (for Id-f)

As disclosed in Figure 2, the triazole derivatives Ia-f were prepared by reacting thiocarbohydrzide with appropriate carboxylic acids. Based on the available reports, this type of reaction has been performed with no need to any solvents or catalysts. In cases that the carboxylic acid is liquid, its excess amount serves both as reagent and solvent. In our study, we prepared derivatives Ia-c using formic acid, acetic acid and propionic acid in excess amounts respectively according to the previous reports (19,20). The acid residue was removed by simply washing the crude product with multiple portions of water. To prepare compounds Id-f, solid carboxylic acids including indole-3-acetic acid, mandelic acid and thiophene-2-acetic acid were employed. The desired derivatives were synthesized in a solvent free condition. Heating equimolar amounts of TCH and the acids at 165-170 °C led to a fused mass and the reaction proceeded to completion in 30 min. It must be noted that the above solvent free methodology was practiced employing substituted benzoic acids which was unfruitful in all cases. This might be due to the fact that aromatic carboxylic acids are less reactive compared to aliphatic ones. In addition, substituted benzoic acids melt at relatively high temperatures in which TCH undergoes decomposition. 

The spectral data of the triazole derivatives were in agreement with the desired structures. In the IR spectra, N-H stretch was observed at about 3300 cm^-1^. In the ^1^H NMR spectra of compounds Id-f, the hydrogens on the aromatic substituents resonated at 6.99-7.54. N_1_-H hydrogen appeared as broad D_2_O exchangeable peaks at 13.6 ppm. It is noteworthy that NH_2_ hydrogens gave two close singlets with unequal integrations at 5.6 ppm in the ^1^H NMR spectra of compounds Ie and If. It could be speculated that the thioamide N_4_ in the triazolethione ring contribute to resonance with the thiocarbonyl group and each peak in the 1H NMR spectra results from one of these possible resonance structures. The molecular mass of the triazole derivatives was confirmed by appearance of mass values corresponding to hydrogen and sodium adducts of the intact molecules.

**Figure 3:**
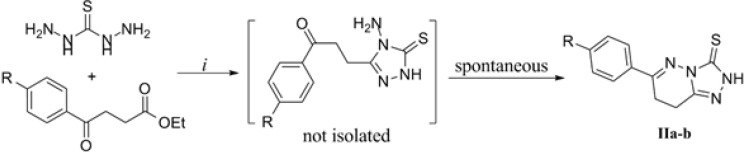
Synthesis of triazolopyridazines IIa-b. The starting ketoesters were prepared by Friedel-Crafts succinylation of benzene (22) or 4-chlorobenzene (23) and conversion of the obtained acids to corresponding ethyl esters (24,25).

The next effort was extending the above methodology to γ-ketoacids. However, the reactions did not lead to the desired triazoles in quantitative yields. In this case, based on the available reports indicating the preparation of some triazoles by reacting TCH with esters of carboxylic acids in a basic medium (21) a modified method was employed. As shown in Figure 3, TCH and ethyl esters of γ-ketoacids were reacted in the presence of methanolic solution of sodium methoxide. The acidic workup after 24 h furnished the triazolopyridazine and not the intermediate triazole products (Figure 3, the compound in brackets). This is supported by the analytical data indicating the formation of triazolopyridazines IIa and IIb as the sole product. In the IR spectra of compounds IIa and IIb, carbonyl stretch band in 1700 region is absent. Furthermore in the mass spectra, molecular masses of the triazolopyiridazine structures (231 as H^+^ adduct of IIa; 265 and 267 as H^+^ adduct of IIb) were present in high abundance while mass values of intermediate triazoles which are 18 units heavier than their corresponding triazolopyridazines were not observed at all. The ^1^H NMR data also conform the proposed structures for IIa and IIb. The presence of second order triplets in ~3.1 ppm (assigned to methylene groups), multiplets (in IIa) or doublet of doublets (in IIb) in the aromatic region (assigned to 6-Aryl hydrogens) and a D_2_O-exchangeable singlet in ~13.6 ppm leaves no doubt in the formation of thiazolopyridazines IIa and IIb.

Based on the above discussions, we report the first synthesis of 6-aryl-7,8-dihydro-[1,2,4]triazolo[4,3-*b*]pyridazine-3-thiones using TCH and ethyl esters of γ-ketoacids by triazole ring closure and intramolecular imine condensation in a single-step strategy. The obtained results deserve further studies on the application of this reaction to synthesize novel heterocyclic compounds and evaluation of their potential bioactivity.

**Figure 4:**
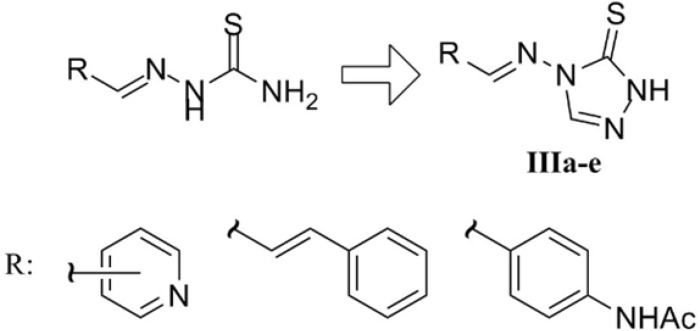
Schiff bases IIIa-e as cyclic analogues of previously reported bioactive thiosemicarbazone derivatives of pyridine-3-carboxaldehyde (26), cinnamaldehyde (27) and 4-acetaimdobenzaldehyde known as thiacetazone (28).

The last series of the synthesized compounds (IIIa-e) were synthesized based on their analogy to bioactive thiosemicarbazones reported in literature. In fact, these derivatives could be regarded as cyclic analogues of thiosemicarbazones disclosed in figure 4 which have been reported to exert antimicrobial and antimycobacterial activities (26-28).

**Figure 5:**
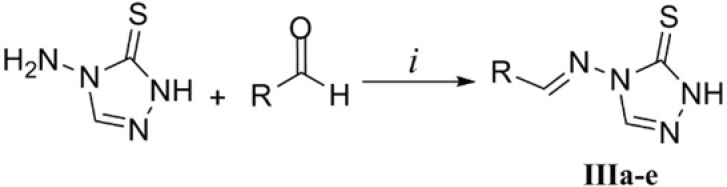
Synthesis of Schiff bases IIIa-e. Reaction conditions: *i*) AcOH, 90 °C.

As disclosed in Figure 5, the desired triazole Schiff bases were prepared by heating compound Ia and appropriate aldehydes in glacial acetic acid according to our previous work (29). This methodology led to the fast synthesis of compounds IIIa-e in good yields. In the ^1^H NMR of the derivatives, the substituted aromatic rings relating to the starting aldehydes could be readily distinguished by their characteristic multiplicity patterns. The traizole C_3_-H and imine H gave rise to two singlets at about 8.9 ppm and 9.5 ppm. Observation of the molecular mass of the Schiff base products further confirmed the structures of compounds IIIa-e.

*Biological activity*

The synthesized derivatives were evaluated for their antimycobacterial activity against *Mycobacterium bovis BCG* and the MIC values have been listed in Table 1. As evident from the MIC values, the triazole derivatives Ia-f exhibited moderate activities and among them compound Ib (R = CH_3_) was the most potent derivative with IC_50_ value of 31.25 µg/mL. While the triazolopyridazine derivative IIa showed a moderate activity (MIC = 62.5 µg/mL), the *p*-chloro substituted compound IIb was found to be inactive. Amongst the compounds IIIa-e, Schiff bases of 2-pyridinecarboxaldehyde (3a) and cinnamaldehyde (3e) exhibited the highest activity in the series with MIC value of 62.5 µg/mL.

**Table 1:** Antimycobacterial activity and cytotoxicity of the synthesized derivatives.

**Compound**	**R**	**Antimycobacterial activity** a ** (24 h/48 h)**	**Cytotoxicity** b ** (48 h)**
		**MIC (µg/mL)**	**IC**_50_ ** (µg/mL)**
***Ia***	H	62.5/62.5	>100
***Ib***	Methyl	31.25/31.25	>100
***Ic***	Ethyl	62.5/62.5	>100
***Id***	(3-indolyl)methyl	62.5/62.5	>100
***Ie***	hydroxy(phenyl)methyl	62.5/62.5	>100
***If***	(2-thienyl)methyl	62.5/62.5	>100
***IIa***	H	62.5/62.5	>100
***IIb***	Cl	>500/>500	>100
***IIIa***	2-pyridyl	62.5/125	>100
***IIIb***	3-pyridyl	125/125	>100
***IIIc***	4-pyridyl	93.75/125	>100
***IIId***	(4-acetamido)phenyl	125/125	>100
***IIIe***	Styryl	62.5/62.5	42.63
**Ethambutol**		0.75/0.75	-
**DMSO**		6.5/6.5 % v/v	-

a Assayed against *Mycobacterium bovis BCG*

b Assayed against Fibroblast L929 cell line

In order to evaluate the potential toxic effects of the synthesized derivatives, MTT assay was performed on Fibroblast L929 cell line. As disclosed in Table 1, the majority of the derivatives were non-toxic at 100 µg/mL. This suggests that the synthesized derivatives have selective toxicity on *Mycobacterium bovis* which means they inhibit mycobacterial growth at concentrations which are non-toxic to normal cells.

Reviewing the literature reveals that many groups by employing the versatility of TSC/TCH in heterocyclic synthesis have prepared different heterocyclic compounds as potential bioactive agents. For instance, in a recent study, conversion of some thiosemicarbazones to 2,4-disubstituted thiazoles resulted in a group of derivatives with potent antibacterial activities against gram negative and gram positive bacterial strains (30). By incorporating the TSC moiety in novel heterocyclic compounds, researchers found some triazole and polycyclic quinoxaline derivatives with significant inhibitory activity against *Entamoeba histolytica* (31,32). Cyclic thiazole and triazole analogues of thiosemicarbazones have received the attention of researchers in a few efforts to develop potential antiviral (33,34) antimicrobial (35,36) and antimycobacterial (6,34,37) agents. 

In light of the above studies along with our previous experiences mentioned earlier, the present work as an attempt to evaluate the bioactivity of different cyclic versions of TSC and TCH seems logical. In this study as a general view, the triazole derivatives Ia-f showed higher activity than the other two series of compounds. This might suggest that 3-substituted triazole derivatives have the best pharmacophoric characters for antimycobacterial activity among the tested compounds. In addition, the cytotoxicity assay proved the safety of these derivatives on Fibroblast L929 as a normal cell line. Compound IIb as the most potent derivative in the current study is a small molecule (MW = 130.17) and hence a suitable starting point to conduct future lead optimization studies aimed at obtaining efficient antimycobacterial compounds.

## Conclusion

In the search for novel cyclic analogues of bioactive thiosemicarbazide derivatives, in this work 13 derivatives divided into three subgroups including 3-substituted 4-amino-1,2,4-triazole-5-thiones (Ia-f), 6-Aryl-7,8-dihydro-[1,2,4]triazolo[4,3-*b*]pyridazine-3-thiones (IIa-b) and Schiff bases of 4-amino-1,2,4-triazole-5-thione (IIIa-e) were synthesized. Triazole derivatives of series I were prepared by heating equimolar mixture of thiocarbohydrazide and different carboxylic acids. Using γ-ketoesters to react to thiocarbohydrazide in a basic medium furnished novel triazolopyridazines of series II. The employed synthetic methodology could be considered as a new route to triazolopyridazines in which both triazole and pyridazine ring is formed in a single step. Finally, compounds of series III were synthesized by heating 4-amino-1,2,4-triazole-5-thione and appropriate aldehydes in glacial acetic acid. After confirming the structures of the synthesized derivatives by different spectroscopic and analytical methods, antimycobacterial activity of the derivatives was assessed against *Mycobacterium bovis BCG*. Among the derivatives, 3-substituted triozles Ia-f were among the most potent derivatives and methyl substituted derivatives Ib exhibited the highest activity with MIC value of 31.25 µg/mL. The derivatives were also tested for their cytotoxicity on Fibroblast L929 as a normal cell line and the majority of the synthesized derivatives were non-toxic at 100 µM. Activity data suggests that 3-substituted 4-amino-1,2,4-triazole-5-thione has the best structural backbone and demands further lead optimization studies to obtain more effective antimycobacterial derivatives.
